# Nonprewhitening model observers in the Fourier and spatial domain: a comparison of predictions for iterative and deep learning reconstruction in computed tomography

**DOI:** 10.1093/rpd/ncaf160

**Published:** 2026-03-13

**Authors:** Gavin Poludniowski, Rebecca Titternes, Jessica Flores, Daniel Thor

**Affiliations:** Department of Nuclear Medicine and Medical Physics, Karolinska University Hospital, Framstegsgatan 23, D1:00, 17164 Stockholm, Sweden; Department of Clinical Science, Intervention and Technology, Karolinska Institutet, Alfred Nobels Allé 8, ANA Futura, 14152 Huddinge, Sweden; Department of Nuclear Medicine and Medical Physics, Karolinska University Hospital, Framstegsgatan 23, D1:00, 17164 Stockholm, Sweden; Department of Clinical Science, Intervention and Technology, Karolinska Institutet, Alfred Nobels Allé 8, ANA Futura, 14152 Huddinge, Sweden; Department of Nuclear Medicine and Medical Physics, Karolinska University Hospital, Framstegsgatan 23, D1:00, 17164 Stockholm, Sweden; Department of Clinical Neuroscience, Karolinska Institutet, Karolinska, Tomtebodavägen 18A Floor 5, 17177 Stockholm, Sweden; Department of Nuclear Medicine and Medical Physics, Karolinska University Hospital, Framstegsgatan 23, D1:00, 17164 Stockholm, Sweden; Department of Oncology-Pathology, Karolinska Institutet, Anna Steckséns gata 30A, D2:04, 171 64 Stockholm, Sweden

## Abstract

The nonprewhitening matched filter (NPWMF) is frequently used to assess task-based image quality in computed tomography (CT). However, modern reconstruction algorithms, based on iterative reconstruction (IR) or Deep Learning image reconstruction (DLIR), exhibit properties that undermine Fourier domain approaches. One alternative is to abandon the NPWMF. Here, instead, calculation of the NPWMF in the spatial domain is explored with and without assumption of Gaussian observer response. Model observer predictions of area-under-the-curve were determined for a Revolution CT scanner (GE Healthcare) and a NAEOTOM Alpha scanner (Siemens Healthineers). For the former, the vendor’s IR and DLIR were investigated. For the latter, the vendor’s IR was used and compared to results from a reader study. Results support the conclusion that Fourier domain calculations can exaggerate benefits of denoising and that spatial domain calculations can provide good agreement with human observers. Assumption of Gaussian observer response did not lead to substantial errors.

## Introduction

Task-based image quality metrics are recommended to evaluate low-contrast detectability in CT systems rather than metrics such as contrast-to-noise ratio [1, 2]. Task-based measures are of particular importance when noise texture or resolution vary between imaging conditions. Task-based assessment requires a clearly defined task, relevant images, or image properties and a well-defined measure of performance. Human observers can assess image quality through receiver operating characteristics (ROC) or multiple-alternative forced choice (M-AFC) experiments [3] or even visual grading studies [4]. Alternatively, mathematical models that simulate human performance can be used. If such model observers are dependable, they can be used for system comparison, or optimization of image quality against radiation dose, without extensive studies with radiologists as readers [5].

The nonprewhitening matched filter (NPWMF) is a model observer frequently used to assess task-based image quality in computed tomography (CT) [1]. It is commonly calculated in the Fourier domain [6–15]. It has been found to correlate well with human performance in certain tasks for reconstruction by filtered backprojection [8, 16]. Modern reconstruction algorithms, however, exhibit properties that can undermine the validity of the typical Fourier domain calculations of this observer. A popular alternative is to select an entirely different model such as the channelized hoteling observer, which is typically calculated in the spatial domain [6, 8]. This raises the question: are the issues with the NPWMF that have been identified for modern reconstruction algorithms due to the mathematical observer itself or merely its calculation using Fourier techniques? This work attempts to answer this question by calculation of the NPWMF in both the spatial and Fourier domains. Calculation of the NPWMF in the spatial domain is not a novel proposal (see, for example, Tapiovaara and Wagner [17]), but it is relatively rarely adopted [16, 18] and even more rarely compared to its Fourier equivalent. Recently, we conducted such a comparison and outlined a procedure for spatial domain calculation in detail [19]. This work extends those results by investigating an additional scanner and reconstruction algorithm and comparing to human observer results. The validity of assuming Gaussian independent observer responses is also investigated.

Results are presented in terms of the area-under-the-curve (AUC)—representing the area-under-the-curve of an ROC study—which is also equivalent to the percentage correct in a 2-AFC study. Results are shown for acquisitions on two scanners: the Revolution CT (GE Healthcare, Waukesha, WI) and the NAEOTOM Alpha photon-counting CT (Siemens Healthineers, Forchheim, Germany). In the former case, results are obtained for the vendor’s iterative reconstruction (IR) and deep learning image reconstruction (DLIR) algorithms with differing levels of noise suppression. In the latter case, results are shown for the vendor’s iterative algorithm with differing strength levels with comparison to human observer data.

## Methods

### Phantoms and CT acquisitions

**Figure 1:**
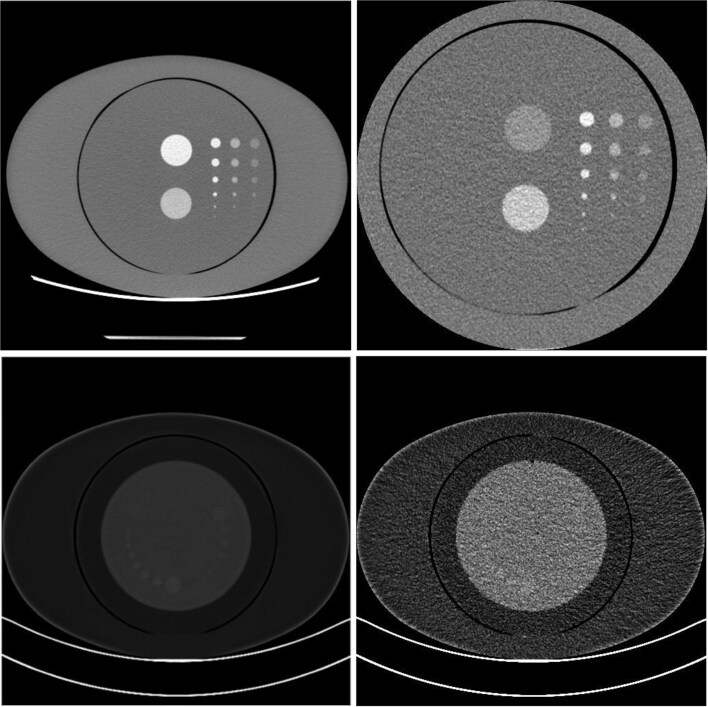
The phantoms used in this study. (a) Displays a CT slice of the in-house phantom combined with the CTP579–15 body annulus, imaged on the GE revolution scanner at higher dose and with a larger reconstruction field-of-view than used for the experiments; (b) provides an example image of the phantom from the image set used in the study; (c) displays the Catphan 600 phantom and body annulus under low noise conditions imaged on the Siemens NAEOTOM alpha scanner; (d) provides an example image of the phantom from the image set used in the study.

CT scan data from two previous studies by our group were used for the analysis [11, 15]. The phantom used for the investigation with the GE Revolution scanner is depicted in Fig. 1a and b. Figure 1a shows an enlarged reconstruction field-of-view to encompass the whole phantom while Fig. 1b shows an example image slice used in the study. The phantom represents an adult abdomen and consists of an in-house manufactured central section of 200 mm diameter composed of water equivalent materials, within an extension ring (CTP579–15 body annulus, The Phantom Laboratory, Salem, New York, USA) of size 250 mm (height) × 350 mm (width). The central section features a set of drilled cavities with varying diameters (1, 2, 4, 6, 8, 10 mm). Two 32 mm diameter features are also included, consisting of a further cavity and a bone substitute. Slabs of the same size as the central section were placed either side of it, so the entire phantom had a 150 mm extent along the z-axis. For this phantom, the features (filled cavities) represent a lesion with iodine enhancement. The cavities were filled with varying concentrations of iodine solution, created by dilution of contrast media.

The phantom used for investigation of the Siemens NAEOTOM Alpha scanner was a Catphan 600 quality assurance phantom (The Phantom Laboratory, Salem, New York, USA) combined with the CTP 579–15 annulus, again to represent an adult abdomen. The CTP515 low-contrast module provided the features of interest. In this case, the features represent subtle contrast between soft-tissues or water. This phantom configuration is depicted in Fig. 1c at a low noise level and in Fig. 1d at a dose level corresponding to the study.

The acquisition and reconstruction parameters for the two experiments are summarized in Table 1. In the case of the Revolution CT experiment, analysis focused on a contrast of 2 mg I/ml and a 2 mm diameter feature. This concentration approximates that in the liver parenchyma in the venous phase [20] and the diameter ensures that there is an appreciable probability of such a feature being missed by an observer. Images were reconstructed with three levels of iterative reconstruction (ASiR-v: 0%, 50%, 100%) and Deep Learning reconstruction (TrueFidelity: Low, Medium, High).

**Table 1:** Scan and reconstruction parameters for acquisitions with two CT scanners.

**Property**	**Units**	**Revolution CT scanner**	**NAEOTOM Alpha scanner**
Tube voltage	kV	120	120
Mode	–	Axial	Spiral
Collimation	mm	40 (64 × 0.625)	58 (144 × 0.4)
Slice thickness	mm	5	1
Rotation time	S	0.5	0.5
Pitch	–	–	0.8
Tube current time	mAs	127	150
CTDI_vol_	mGy	4.8	10
DFOV	mm	240	350
Kernel	–	Standard	Br40
Reconstruction	–	ASiR-v: 0%, 50%, 100% TrueFidelity: L, M, H	QIR: off, 2,4
Reconstruction monoenergy	keV	–	67
Repeat scans	–	110	29
Slices per scan	–	5	48

In the NAEOTOM Alpha experiment, analysis focused on the 1% contrast supra-slice targets (~10 HU) and features of size 2–9 mm. Images were reconstructed with three level of quantum iterative reconstruction (QIR: off, 2, 4).

### Human observer experiments

Results from a previous four-alternative forced choice experiment (4-AFC) with human readers were available for feature sizes of 4, 6, and 9 mm and QIR levels 2 and 4 [15]. The AFC study involved five readers, including four physicists and one radiologist. A custom graphical user interface based on MATLAB (R2022a, The MathWorks Inc., Massachusetts, United States) was used for the study. Participants were coached on how to conduct the study and had an opportunity to practice on an independent set of images prior to commencing the study. A fixed window of 140 HU was used for display on a radiological monitor and signal present and absent images were cropped to the same size. Per feature size and QIR level, 650 trials were completed for each participant. AUC was calculated from percentage correct (PC) using tabulations for Gaussian independent responses [21]. Uncertainties were estimated as the standard error on the mean across participants. Further details are available in Flores *et al.* [15].

### Model observer calculations

The model observer (MO) of interest is the nonprewhitening matched filter (NPWMF). An image will be represented as a column vector, ${\mathbf{i}}_{h,i}$, where $h=1$ or 2 for the signal absent or present cases, respectively, and *i* is label for the particular image instance. The NPWMF decision variable for an image is a scalar variable defined as [5],


1 \begin{eqnarray*} {L}_{h,i}=\mathbf{\Delta }{\overline{\mathbf{i}}}^{\mathrm{T}}\ {\mathbf{i}}_{h,i}, \end{eqnarray*}

where the “T” superscript represents the transpose operation and $\mathbf{\Delta }\overline{\mathbf{i}}$ is the template vector corresponding to the expected value of the difference between the image vector in signal present and absent cases. This can be expressed as:


2 \begin{eqnarray*} \Delta \overline{\mathbf{i}}=\mathrm{E}\left[{\mathbf{i}}_{h=2}-{\mathbf{i}}_{h=1}\right]=\mathbf{t}\Delta \mathbf{f}, \end{eqnarray*}

where E[…] denotes the expected value, $\mathbf{\Delta }\mathbf{f}$ is the known difference in the object itself in the signal present and absent cases multiplied by a spatial transfer function, $\mathbf{t}$. The template can therefore either be estimated directly based on a very large ensemble of noisy images or on knowledge of the object and the spatial transfer of the system.

The AUC in an ROC study (or equivalently percentage correct in a 2-AFC study) can be estimated from the decision variables for a set of images. For a set of *n*_1_ signal absent and *n*_2_ signal present images, the AUC is [22],


3 \begin{eqnarray*} \mathrm{AUC}=\frac{1}{n_1{n}_2}\sum_{i=1}^{n_1}\sum_{j=1}^{n_2}H\left({L}_{2,i}-{L}_{1,j}\right), \end{eqnarray*}

where *H* is the Heaviside step function. A Gaussian internal noise term can be added to model the inefficiency of the human observer, in which case Eq. (3) becomes a convolution:


4 \begin{eqnarray*} \mathrm{AUC}&=\frac{1}{n_1{n}_2}\sum_{i=1}^{n_1}\sum_{j=1}^{n_2}H\ast{\mathcal{N}}_{\sigma}\left({L}_{2,i}-{L}_{1,j}\right)\nonumber\\&=\frac{1}{n_1{n}_2}\sum_{i=1}^{n_1}\sum_{j=1}^{n_2}\mathrm{\phi} \left(\frac{L_{2,i}-{L}_{1,j}}{\sigma}\right), \end{eqnarray*}

where ${\mathcal{N}}_{\sigma }$ is the zero-mean normal distribution with a standard deviation of $\sigma$ and $\mathrm{\phi}$ is the cumulative normal distribution.

The signal-to-noise of the decision variable is also a figure-of-merit and often equated with a detectability index (*d’*). This can be expressed as [5],


5 \begin{eqnarray*} d{\prime}^2=\frac{{\left(\mathrm{E}\left[{L}_{2,i}\right]-\mathrm{E}\left[{L}_{1,i}\right]\right)}^2}{\frac{1}{2}\left(\mathrm{Var}\left[{L}_{1,i}\right]+\mathrm{Var}\left[{L}_{2,i}\right]\right)}, \end{eqnarray*}

where E[…] and Var[…] denote the expected value and variance, respectively. In the spatial and Fourier domains, the detectability index can be related as,


6 \begin{eqnarray*} d{\prime}^2=\frac{{\left(\mathbf{\Delta }{\mathbf{f}}^{\mathrm{T}}\ {\mathbf{t}}^{\mathrm{T}}\ \mathbf{t}\mathbf{\Delta }\mathbf{f}\right)}^2}{\mathbf{\Delta }{\mathbf{f}}^{\mathrm{T}}\ {\mathbf{t}}^{\mathrm{T}}\ \mathbf{n}\ \mathbf{t}\mathbf{\Delta }\mathbf{f}}=\frac{{\left[\int d\boldsymbol{\nu}\ {\left|\mathbf{\Delta }\mathbb{F}\left(\boldsymbol{\nu} \right)\right|}^2\ {\left|\mathbb{T}\left(\boldsymbol{\nu} \right)\right|}^2\right]}^2}{\int d\boldsymbol{\nu}\ {\left|\mathbf{\Delta }\mathbb{F}\left(\boldsymbol{\nu} \right)\right|}^2\ {\left|\mathbb{T}\left(\boldsymbol{\nu} \right)\right|}^2\ \mathbb{N}\left(\boldsymbol{\nu} \right)} \end{eqnarray*}

where **n** is the average noise covariance matrix and the large-case “double-struck” symbols represent Fourier transformed quantities. Note that the spatial domain representation has been expressed in terms of matrix operations while the Fourier domain representation has been expressed in terms of integrals over the 2D spatial frequency, $\boldsymbol{\nu}$. In the above equation, 𝕋 is the modulation transfer function (MTF) and $\mathbb{N}$ is the noise power spectrum (NPS). In the Fourier approach to calculation of the model observer, the MTF and NPS can be determined from phantom images and then used to estimate *d’* for an arbitrary selected feature ($\mathbf{\Delta }\mathbf{f}$) [2].

In a previous work [19], we detailed a procedure to estimate the numerator and denominator using regions-of-interest (ROIs) and repeat imaging, without explicit estimation of the input signal ($\mathbf{\Delta }\mathbf{f}$), spatial transfer characteristics ($\mathbf{t}$) o,r noise covariance ($\mathbf{n}$). For *n* repeat scans and *m* usable slices per scan, the percentage uncertainty (one standard deviation) in such an estimation of *d’* can be estimated as,


7 \begin{eqnarray*} u=100\sqrt{\frac{1}{nm}\frac{1}{d{\prime}^2}+\frac{1}{4}\frac{1}{\left(n-1\right)m}} .\end{eqnarray*}

This assumes one feature present and one feature absent region-of-interest per image slice and independent responses.

The equality of Eq. (6) between the spatial and Fourier expressions is only valid for stationary noise. If the MTF is calculated for an appropriate location and contrast level—referred to then as a task-transfer function (TTF)—and the noise can be assumed stationary over a local region—referred to as quasi-stationary—then the calculations in the two domains may remain closely equivalent. This agreement can still break down as the strength of denoising is increased in modern reconstruction algorithms [8].

To obtain AUC from d’, Gaussian independent responses are usually assumed [3], for which,


8 \begin{eqnarray*} \mathrm{AUC}=\mathrm{\phi} \left(\frac{d^{\prime }}{\sqrt{1+\beta }}\frac{1}{\sqrt{2}}\right), \end{eqnarray*}

where $\mathrm{\phi}$ is again the cumulative normal distribution function and $\beta$ is an internal noise parameter used to degrade model observer performance to the efficiency of human observers. The Gaussian responses assumption is expected to be approximately valid even for non-Gaussian image noise due to the Central Limit Theorem, as the mathematical observer involves a summation over an area of an image [1]. As de-noising strength of a reconstruction algorithm is increased, however, a deterioration in the approximation might be expected.

When Gaussian responses can be assumed, Eq. (8) is equivalent to Eq. (4) when,


9 \begin{eqnarray*} {\sigma}^2=\beta \left(\mathrm{Var}\left[{L}_{1,i}\right]+\mathrm{Var}\left[{L}_{2,i}\right]\right). \end{eqnarray*}

Three methods to estimating the NPWMF AUC will be explored in this work:

(1) Fourier calculation using Eqs. (6) and (8) based on the TTF and NPS, assuming quasi-stationarity and Gaussian responses
(2) Spatial domain calculation for a set of images using Eqs. (1–2), (5) and (8) assuming Gaussian responses but not requiring quasi-stationarity
(3) Spatial domain calculation for a set of images using Eqs. (1–2) and (4) assuming neither Gaussian responses nor quasi-stationarity

The first approach is that most typically adopted for recent the NPWMF in CT studies. The second two test the validity of two assumptions (stationarity and Gaussian responses).

### Implementation of model observers

For the Fourier domain method, AUC was calculated for the different scanners, features, and reconstructions according to the approach outlined in the previous subsection; greater detail on the methodology is available in our previous publications [11, 15].

**Figure 2:**
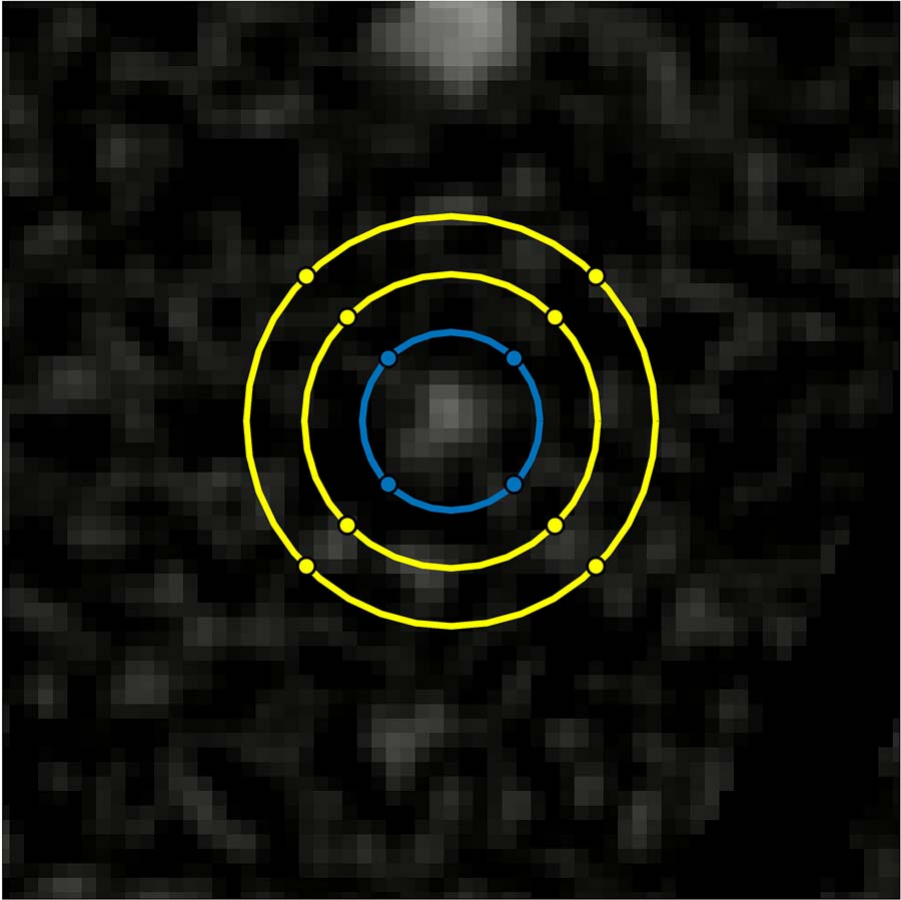
An illustration of the placement of regions-of-interest ROIs around a 2 mm diameter feature of the in-house phantom. The central blue circle define a feature-present ROI encompassing the feature of interest. The outer annulus described by the yellow border defines pixels used for determining local background level (averaged over pixel and repeats scans).

The spatial domain calculations also followed that described in an earlier publication [19]. Feature ROIs were placed over the relevant features with a size equal to their radius expanded by *N*_exp_ pixels. A local background level was determined from the mean value in an annulus surrounding the feature (*N*_ann_ pixels wide), averaged over all repeat scans. A gap was employed between the feature ROI and the background annulus (*N*_gap_ pixels). This is illustrated in Fig. 2. For the in-house phantom the following values were used: *N*_exp_ = *N*_ann_ = *N*_gap_ = 4. For the Catphan phantom the following values were used: *N*_exp_ = *N*_ann_ = *N*_gap_ = 3. Any pixels in a background annulus that overlapped with the ROI of a neighboring feature were excluded from calculations. This was only relevant for the Catphan phantom, due to the close proximity of the different sized features (see Fig. 1c). Feature-absent ROIs were placed with the same size as the feature-present ROIs. For the in-house phantom (GE Revolution experiments), these ROIs were places in the left–right mirror positions to the feature-present ROIs, while for the Catphan phantom (NAEOTOM Alpha) they were placed in the background material in close proximity to the feature-present ROIs.

A value of $\beta =3.0$ was selected for the internal noise parameter. This was chosen to match spatial domain predictions of AUC using Eq. (4) to the human observer data used in this study. Human data was available for the NAEOTOM Alpha images and quantum iterative reconstruction levels 2 and 4 (QIR-2, QIR-4) [15].

Estimates of uncertainty on AUC are challenging to quantify for the standard Fourier-based method in this work (i.e. “method 1”). They were only estimated for the GE Revolution data for which the TTF was available for six slices and the NPS for 110 repeat scans. The 95% confidence interval on AUC was determined by the percentile bootstrap technique with 1000 resamples of the average TTF and NPS over slices and repeats. Uncertainty of spatial domain estimates of AUC via d’ (i.e. “method 2″) were simply calculated using Eq. (7). For spatial domain estimates directly from decision variables (i.e. “method 3″), the AUC was estimated for each slice location (z-axis position) and the standard error on the mean across slices used as an uncertainty estimate.

## Results

For the GE Revolution acquisitions, AUC predictions for the 2 mm diameter feature (with 2 mg I/ml) are presented in Table 2. In all cases, the two spatial domain methods provided equivalent results, as assessed by overlapping CIs. For the lowest level of IR—expected to most closely resemble traditional filtered backprojection reconstruction—the spatial and Fourier calculations are also in close agreement with the Fourier prediction lying inside the CI of the spatial domain value. As the IR strength is increased, Fourier calculations indicate a small but consistent increase in AUC. The reverse trend is seen for spatial domain calculations (although the change is marginal). For the strongest IR level, the Fourier prediction is ~3% higher than the spatial domain predictions. Similar results can be noted for DLIR, although in this case the Fourier domain predictions never lie within the CIs of the spatial domain values.

**Table 2:** Area-under-the-curve (AUC) values calculated using Fourier and spatial domain approaches for the GE revolution CT data.

**Reconstruction**	**AUC**		
	**Fourier Estimate**	**Spatial Estimate, Gaussian response (LL, UL)**	**Spatial Estimate, general (LL, UL)**
ASiR-v 0%	0.833 (0.832,0.835)	0.838 (0.825, 0.851)	0.841 (0.833, 0.848)
ASiR-v 50%	0.844 (0.843,0.846)	0.832 (0.819, 0.844)	0.836 (0.830, 0.842)
ASiR-v 100%	0.853 (0.852, 0.855)	0.818 (0.805, 0.831)	0.827 (0.822, 0.833)
TrueFidelity Low	0.868 (0.866, 0.870)	0.847 (0.834, 0.859)	0.850 (0.843, 0.857)
TrueFidelity Medium	0.880 (0.878, 0.882)	0.849 (0.836, 0.861)	0.852 (0.845, 0.859)
TrueFidelity High	0.893 (0.891, 0.895)	0.851 (0.838, 0.863)	0.855 (0.848, 0.861)

The AUC corresponds to a 2 mm diameter feature and a contrast of 2 mg I/ml. Results are presented for three levels of iterative reconstruction (ASiR-v) and three levels of Deep Learning image reconstruction (TrueFidelity). Estimates of the lower limit (LL) and upper limit (UL) of the 95% confidence interval are provided. Spatial domain estimates are provided assuming both Gaussian responses and in the general case.

**Figure 3:**
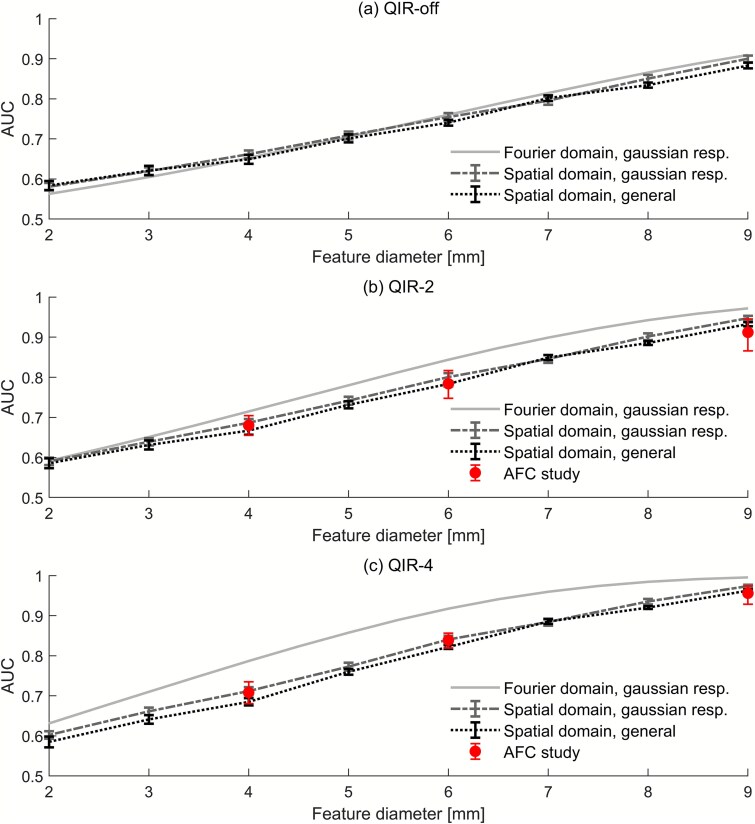
Fourier and spatial domain predictions of the area-under-the-curve (AUC) for the NAEOTOM alpha scanner with 10 HU contrast and various feature sizes and three reconstruction selections: (a) QIR off, (b) QIR level 2, and (c) QIR level 4. Human reader results derived from an alternative forced choice (AFC) study are included for QIR-2 and QIR-4 (filled circles). Fourier domain results are presented with assumption of Gaussian observer responses (solid lines). Spatial domain results are presented both with (dashed) and without (dotted) assumption of Gaussian responses.

The AUC results for the Siemens NAEOTOM Alpha acquisitions are presented in Fig. 3. For the QIR-off reconstruction (see Fig. 3a) there is close agreement between Fourier and both spatial domain methods across the range of feature sizes. This is expected, as QIR-off is equivalent to weighted filtered backprojection for the monoenergy value used in this study [23]. However, as the QIR strength is increased (see Fig. 3b and c) differences emerge. The predictions of the two spatial domain methods remain close, albeit with a small but growing discrepancy with increasing QIR level. Both spatial domain calculations also agree well with the human observer results (available for QIR-2 and QIR-4, only) for the selected value of internal noise parameter ($\beta =3.0$). However, although the Fourier domain estimates correctly predict improved performance with increased QIR strength, when using the same value of internal noise parameter as for the spatial domain, they overestimate the extent (see Fig. 3c).

## Discussion

Previous publications have compared the nonprewhitening matched filter observer in the spatial domain to the channelized Hotelling observer in the spatial domain [9], or the NPWMF in the Fourier domain to the Hotelling observer [16]. In this work we have investigated a single observer—the NPWMF—in both domains. This builds on a recent publication [19], however there are several novel features. Firstly, focus is exclusively on features for which an observer has an appreciable chance of missing (AUC < 0.9). Secondly, observations are extended to an additional (photon-counting) CT scanner with a different IR algorithm. Thirdly, predictions are compared to human observer results. Fourthly, the impact of the assumption of Gaussian observer responses is investigated.

It has been known for at least a decade that the NPWMF observer can lead to inflated estimations of the performance of IR [1, 8]. The results of this study confirm this, while extending it to Deep Learning reconstruction. However, they also indicate that the problem may not be the NPWMF observer itself but rather its approximation in the Fourier domain. Certainly, the agreement of the spatial domain NPWMF estimates with the human observer results in this study is excellent, regardless of whether the assumption of Gaussian observer response is applied (“method 2”) or not (“method 3”). However, the Fourier calculations (“method 1”) diverged from the spatial domain predictions as the iterative strength was increased. Since the NPWMF is defined in the spatial domain and the Fourier calculation is an approximation, if the two calculations disagree then the Fourier calculation is not, it can be argued, accurately providing the NPWMF observer at the location of the feature.

Despite known limitations of the Fourier approach it remains much in use in CT [10–15], with many authors using the NPWMF family of model observers and assuming that use of a contrast-dependent MTF (i.e. TTF) is sufficient to account for the effects of non-linearity in reconstruction. The results presented here suggest that more caution should be exercised in adopting such an approach, particularly if assessing the benefit of increasing noise suppression level in a reconstruction algorithm. Adopting or comparing to a spatial domain approach—whether with the NPWMF or the channelized Hotelling observer—would be a preferable option.

It should be noted that the Fourier predictions of this work could have been normalized to better fit the human data at high iterative strength by using a larger value of the internal noise parameter. Indeed, Flores *et al.* did this with the same observer data in an earlier study [15], which lead to a reasonable description of reader results over the limited range of reconstruction settings investigated in that study (QIR 2–4). Choosing different internal noise parameter values for Fourier and spatial domain calculations would, however, introduce discrepancies between spatial domain and Fourier domain predictions for the QIR-off (and ASiR-v 0%) reconstructions, where they should be in close agreement. Unfortunately, AFC data were not available for the QIR-off case to verify that spatial domain predictions remain in agreement with human reader data.

Other limitations of this study include the limited number of CT scanners and scan conditions/reconstructions investigated. Finally, we note that caution should be applied in assuming that model observers that reproduce human performance in simple phantoms extend to in vivo imaging, given that the signal, background, and location are rarely known exactly, and, the evidence that human can adapt their search template in the presence of certain kinds of anatomical noise [3]. None the less, the nonprewhitening matched filter is an established figure-of-merit for comparing CT systems [2] and it is important that it can be calculated reliably.

## Conclusion

Model observers such as the nonprewhitening matched filter are frequently used for assessing system performance in CT. Fourier domain representations of model observers are known to be valid only for stationary noise. It is common for authors to assume that the use of a contrast-specific task-transfer function and the invoking of quasi-stationarity of noise are sufficient to continue to apply the Fourier domain methodology to modern reconstruction algorithms. The results of this work suggest that this can lead to inflated predictions of the benefit of noise suppression and that spatial domain calculations of the nonprewhitening observer seem to be in good agreement with human observers.
